# Annual Commencement of the Medical Department of the University of Buffalo

**Published:** 1872-02

**Authors:** 


### Annual Commencement of the Medical Department of the University of Buffalo.

The graduating class received their examination before the Curators Tuesday morning, February 20th, which was concluded to the entire satisfaction of all. After the examination was closed all retired to the Hall where a bounteous repast was awaiting them, which was partaken of by all amid general good cheer. At the conclusion of the dinner speeches were made by some of the Professors and Curators. Dr. T. D Strong, of Westfield, President of the Alumni, made a few well chosen remarks to the graduating class, he also reported progress in ascertaining the residences and particulars concerning the Alumni, as the Secretary, Dr. W. W. Miner, was unable to be present. At the conclusion of Dr. Strong's remarks the resignation of Dr

W. W. Miner as Secretary was read and reluctantly accepted. Dr. W. C. Phelps, of the class of 1866, was elected to fill his place, Dr. Strong being unanimously re-elected as President.

At half past seven o'clock the graduating class entered St. James Hall and took seats immediately in front of the stage, which was occupied by Hon. Millard Fillmore, Chancellor of the University, the Faculty and a large number of the Curators and Council of the University, Rt. Rev. A. C. Coxe, and Rev. Wolcott Calkins. Wahle's orchestra was in attendance, and pleasantly varied the exercises with music.

The opening prayer was made by Bishop Coxe, at the conclusion of which Prof. Miner read the following report of the proceedings of the Council:

By recommendation of the Faculty and Curators, the Council of the University have this day voted to confer the degree of Doctor in Medicine upon the fnllowing gentlemen:

| | |
|---|---|
| George Washington Earle, - - | DeRuyter, Madison Co., N. Y. |
| George Rightmire, - - - | Jacksonville, Tompkins Co., N. Y. |
| Elwood Daniel Meeder, - - | Westfield, Chautauque, Co., N. Y. |
| Peter Manuel Wise, - - - - - | Clarence, Erie Co., N. Y. |
| Franz Emil Louis Brecht, - - - - - | Buffalo, Erie Co., N. Y. |
| John Sandford Halbert, - - - - - | Buffalo, Erie Co., N. Y. |
| Don J. Lathrop, - - - - - - | Elma, Erie Co., N. Y. |
| William Montgomery Mercer, - - - | Corunna, DeKalb Co., Ind. |
| Edwin Ruthven Johnston, - - - - - | Dresden, Ontario. |
| Fairfield Snyder, - - - | West Sparta, Livingston Co., N. Y. |
| Horace Grant Hopkins, - - - | Williamsville, Erie Co., N. Y. |
| Justinian K. Piersol, - - - - | Adrian, Lenawee Co., Mich. |
| John Sardis Stearns, - - - - | Andover, Allegany Co., N. Y. |
| Isaac Eugene Bennett, - - - | Angellica, Allegany Co., N. Y. |
| Frank Herman Moyer, - - | Mount Morris, Livingston Co., N. Y. |
| Henry Wilson, - - - - | Westfield, Chautauqua Co., N. Y. |
| William Mason Boddy, - - | Wethersfield, Wyoming Co., N. Y. |
| Charles T. Dildine, - - - - | Dansville, Livingston Co., N. Y. |
| Maxwell G. Walkinshaw, - - - - | Batavia, Genesee Co., N. Y. |
| William C. Raymond, - - - - | Cambria, Niagara Co., N. Y. |
| Ziba Hoyt Evans, - - - - | Ransomville, Niagara Co., N. Y. |
| Charles Benjamin Knowlton, - - - | Buffalo, Erie Co., N. Y. |
| Edward Mulheron, - - - - | Canandaigua, Ontario Co., N. Y. |
| Peter Worp Van Peyma, - - - - | Lancaster, Erie Co., N. Y. |
| Harley Leonard Atwood, - - | Collins Centre, Erie Co., N. Y. |
| Robert Hebenstreit, - - - - - | Buffalo, Erie Co., N. Y. |
| Robert Woolston Smith, - - - | Shelby, Orleans Co., N. Y. |
| Cassius Wallace Gould, - - - - | Batavia, Genesee Co., N. Y. |
| David Vacton Stranahan, Jr., - - - | Warren, Warren Co., Pa. |
| William Albert Wasson, - - - - - | Buffalo, Erie Co., N. Y. |

Royal Ezra Cochrane, - - - - Waterport, Orleans Co., N. Y.
Laird N. Woods, - - - - - - Wheatland, Mercer Co., Pa.
Nathan Porter Johnson, - - - Newfane, Niagara Co., N. Y.
Ahaz Daniel Robbins, - - - - Corning, Steuben Co., N. Y.

The Faculty and Curators deem a thesis upon "Forensic Medicine," by Charles Benjamin Knowlton, as worthy of honorable mention and publication. The following theses are also deemed worthy of honorable mention: "Acute Pneumonitis," by Elwood Daniel Meeder; "Conservatism in Surgery," by Franz Emil Louis Brecht; "Acute Articular Rheumatism," by Henry Wilson; "Hygiene and Stimulants in Fevers," by Justinian K. Piersol. Many others are of unusuai merit, and all show commendable care and thought.

Succeeding the reading of the report of the Council, and after music by the orchestra, the graduates were summoned in detachments to the stage, where they received from the hands of the Chancellor, Hon. Millard Fillmore, their diplomas.

The address to the graduates was delivered by Prof. James P. White, and was listened to with attention and interest. The exercises were closed by the benediction, pronounced by Rev. Wolcott Calkins.

